# COVID-19 vaccination perceptions and intentions of maternity care consumers and providers in Australia

**DOI:** 10.1371/journal.pone.0260049

**Published:** 2021-11-15

**Authors:** Zoe Bradfield, Karen Wynter, Yvonne Hauck, Linda Sweet, Alyce N. Wilson, Rebecca A. Szabo, Vidanka Vasilevski, Lesley Kuliukas, Caroline S. E. Homer

**Affiliations:** 1 School of Nursing, Curtin University, Bentley, Western Australia, Australia; 2 Department of Nursing, Midwifery Education and Research, King Edward Memorial Hospital, Subiaco, WA, Australia; 3 School of Nursing and Midwifery, Deakin University, Burwood, Victoria, Australia; 4 Centre for Quality and Patient Safety Research, Western Health Partnership, Albans, VIC, Australia; 5 Maternal, Child and Adolescent Health Program, Burnet Institute, Melbourne, VIC, Australia; 6 Department of Medical Education, Department of Obstetrics and Gynaecology and Department of Critical Care, Melbourne Medical School, The University of Melbourne, Parkville, VIC, Australia; 7 Gandel Simulation Service, The Royal Women’s Hospital, Parkville, VIC, Australia; Texas A&M University College Station, UNITED STATES

## Abstract

**Introduction:**

Vaccination against COVID-19 is a key global public health strategy. Health professionals including midwives and doctors support and influence vaccination uptake by childbearing women. There is currently no evidence regarding the COVID-19 vaccination perceptions and intentions of those who receive or provide maternity care in Australia. The aim of this study was to address this gap in knowledge and explore the perceptions and intentions regarding COVID-19 vaccination from consumers and providers of maternity care in Australia.

**Methods:**

A national cross-sectional online study conducted in early 2021 in Australia, a country that has had a very low number of COVID-19 cases and deaths. Recruitment was undertaken through parenting and health professional social media sites and professional college distribution lists. A total of 853 completed responses, from women (n = 326), maternity care providers including doctors (n = 58), midwives (n = 391) and midwifery students (n = 78).

**Findings:**

Personal intention to be vaccinated ranged from 48–89% with doctors most likely and women least likely. Doctors and midwifery students were significantly more likely to recommend the vaccine to pregnant women in their care than midwives (p<0.001). Fewer doctors (2%) felt that women should wait until breastfeeding had concluded before being vaccinated compared with 24% of midwives and 21% of midwifery students (p<0.001). More than half of the midwives (53%) had concerns about the COVID-19 vaccine for the women in their care compared with 35% of doctors and 46% of midwifery students. Despite national guidelines recommending vaccination of breastfeeding women, 54% of practitioners were unlikely to recommend vaccination for this group.

**Conclusion:**

This is the first study to explore the perceptions and intentions regarding COVID-19 vaccination from the perspective of those who receive and provide maternity care in Australia. Findings have utility to support targeted public health messaging for these and other cohorts.

## Introduction

A key strategy in the global response to end the global COVID-19 pandemic is vaccination even in countries with low infection rates like Australia. In Australia, a public health program to provide vaccination against COVID-19 commenced in February 2021 [1]. A phased approach was adopted with initial roll-out to priority and high-risk groups including those working in quarantine services, border control, aged care and health sectors, followed by a broader population approach commencing with older people, adults with co-morbidities, and those from Aboriginal and Torres Strait Islander backgrounds [2].

Global regions that have experienced higher prevalence of COVID-19, such as the United Kingdom (UK) [3] and the United States (US) [4], adopted recommendations encouraging women to consider vaccination in pregnancy owing to the risk for severe illness. In Australia, recent updates to guidelines (v5) have also recommended routine COVID-19 vaccination for pregnant women [5] following evidence that the vaccination in pregnancy is safe for pregnant and breastfeeding women [6–8].

Health professionals, including doctors, midwives and students are strongly encouraged to be vaccinated, although this is not mandatory. There is currently no national data on COVID-19 vaccine intention or uptake by health professionals in Australia. Individual practitioner participation and perceptions of vaccination are highly relevant factors for the provision of guidance and advice to the individuals they care for [9]. Health professionals (especially midwives and doctors) play an important role in educating women about potential risks and benefits of vaccination which is likely to influence vaccine uptake, particularly during pregnancy although to what extent this is true for COVID-19 vaccines is unclear [10–12]. Australia is in a unique position globally having had a low rate of COVID-19 infection and death compared to other countries. The country closed international borders in March 2020 and state and territory borders are often closed during outbreak management. Most parts of the country have, by international comparisons, lived relatively COVID-19-free with few public health measures in place to interrupt daily life. Intention to be vaccinated has wavered through the pandemic.

A survey of 2,174 Australian residents in the early stage of the pandemic (March 2020) found that worry about exposure to COVID-19 was a consistent predictor of higher levels of vaccine intentions [13]. In April 2020, 14.2% respondents in a survey of 4000 Australian adults said they were unsure or unwilling to accept a COVID-19 vaccine when it became available [14]. In June 2020, an analysis of parents from the first survey showed that nationally, hesitancy to accept a COVID-19 vaccine had increased by 10% (14.2% in April to 24.2% [95% CI 7.9–12.1]; p<0.0001) [15]. More recently, a different survey of more than 3000 people conducted from August 2020 until April 2021 showed that only just over half would have a COVID-19 vaccine. Negative reporting in the media, uncertainty, particularly in relation to the AstraZeneca vaccine, and changes in policy, recommendation and vaccine availability are likely responsible for the increase in vaccine hesitancy [16].

The intention or uptake of COVID-19 vaccination by women who are planning pregnancy, are pregnant, or currently breastfeeding in Australia is not known. Similarly, the support for COVID-19 vaccination among the maternity health care workforce who care for childbearing women or are pregnant themselves, is not known. Therefore, the aim of our study was to explore COVID-19 vaccination perceptions and intentions of consumers and providers of maternity care in Australia.

## Methods

A cross-sectional exploratory design was used to survey five key cohorts of consumers (women of childbearing age and partners) and providers of maternity care in Australia (midwives, doctors who provide maternity care and midwifery students). This cross-sectional approach provided information from a range of participants at a discrete point in time [17]. We have successfully used this design in previous research on experiences of providing and receiving maternity care during the COVID-19 pandemic in these cohorts [18, 19]. Human research ethical approval was granted by Curtin University (HRE2020-0210) with reciprocal approval issued through Deakin University (2020–175) and The University of Melbourne (2057065).

### Research setting

The survey was released online through social media and professional organisations for 6 weeks from 22 March 2021. At the time of survey release, the V2 national guidelines recommended that pregnant and breastfeeding women with co-morbidities should consult with their doctor to consider whether the vaccine would be appropriate for them in pregnancy [2]. Table 1 indicates the cumulative number of COVID-19 cases, deaths and vaccinations administered in Australia on the start and finish dates of the survey within the whole population [20]. For global context, the comparison data for the (US) and UK (22) are also presented (Table 1). These data highlight the unique situation in Australia and hence the importance to better understand vaccine acceptance in a low infection population.

**Table 1:** Comparison of cumulative cases, deaths and vaccinations between Australia, US and UK [20–23].

	22 Mar 2021			3 May 2021		
	Australia	US	UK	Australia	US	UK
Cumulative Cases	29,203	29·9 M	4·3 M	29,863	32·5 M	4·4 M
Total Deaths	909	543,000	126,588	910	577, 900	127, 588
Vaccinations	281,549	463 M	28·3 M	2·3 M	1·117 B	34·7 M

### Recruitment and sampling

Convenience sampling was used which is an accepted strategy in cross-sectional methods when data are required to be collected in a timely way or in response to time-sensitive phenomena under study as is the case with the COVID-19 pandemic [24]. Recruitment occurred via online social media survey flyers with links directly to the online qualtrics survey were distributed via social media pages of groups with interests such as parenting, midwifery, primary care and obstetrics. Invitations were also sent via the relevant professional colleges including the Australian College of Midwives (ACM) and the Royal Australian New Zealand College of Obstetricians and Gynaecologists (RANZCOG). Women of childbearing age and their partners, midwives, all doctors providing maternity care, and midwifery students were eligible to participate.

### Data collection

Owing to the novel nature of this study and the rapid change in global vaccination strategies and recommendations, there was no existing validated survey tool available to facilitate exploration of maternity consumers’ and care providers’ perceptions and intentions around the COVID-19 vaccine. A survey tool to facilitate exploration of both maternity consumers’ and care providers’ perceptions and intentions regarding the COVID-19 vaccine was developed by the research team. The team is comprised of a national collaboration of clinicians, academics, researchers, and professional leaders with backgrounds in midwifery, obstetrics, public health and psychology and have expertise in both survey design and content expertise. The survey was piloted with up to five stakeholders from each of the cohorts for face validity and clarity.

The online survey was hosted on the online system Qualtrics. An information form was available on the front page of the survey and completion implied consent. Demographic data were collected and then participants were asked a series of fixed-choice questions about their perceptions and intentions regarding the COVID-19 vaccine (see supporting information for the full survey, S1 File). The overall completion rate for those who commenced the survey was 97% indicating a high level of tool acceptability.

### Data analysis

Data were imported into IBM SPSS Statistics v26. Syntax was used to exclude data from participants who did not progress beyond demographic details (n = 22). A total of 17 responses were received from partners; these responses were removed prior to analysis due to the small numbers. Descriptive statistics were used for the demographic data. Chi-square tests were performed identifying differences in responses between women, midwives, doctors, and midwifery students on each survey item. To adjust for multiple tests and reduce the chance of making Type 1 errors, statistical significance was set at p<0.001.

## Results and discussion

A total of 853 completed survey responses were received with responses from each of the eight Australian states and territories. Most respondents indicated English as their primary language; almost three percent spoke another language at home, and three percent identified as Aboriginal or Torres Strait Islander, Australia’s First Nations peoples. Each of the cohorts included people who were pregnant or breastfeeding. One in five (19%) women were currently pregnant and almost one-third (32%) were breastfeeding. Just over half (56%) indicated they were definitely or potentially planning a pregnancy within the next 2 years (Table 2).

**Table 2:** Demographic characteristics of survey participants.

Demographic Characteristics	Women	Medical practitioners	Midwives	Midwifery students
	N = 326	N = 58	N = 391	N = 78
Australia state of residence				
WA	74 (23.5%)	6 (10.5%)	62 (16.2%)	33 (50 0%)
NSW	62 (19.7%)	14 (24.6%)	120 (31.3%)	13 (19.7%)
VIC	88 (27.9%)	24 (42.1%)	66 (17.2%)	7 (10.6%)
QLD	37 (11.7%)	5 (8.8%)	72 (18.8%)	6 (9.1%)
SA	26 (8.3%)	3 (5.3%)	48 (12.5%)	3 (4.5%)
ACT	22 (7.0%)	1 (1.8%)	9 (2.3%)	2 (3.0%)
NT	2 (0.6%)	1 (1.8%)	5 (1.3%)	1 (1.5%)
TAS	4 (1.3%)	3 (5.3%)	1 (0.3%)	1 (1.5%)
Age (years)				
18–25	17 (5.4%)	0	32 (8.4%)	15 (22.7%)
26–30	72 (22.8%)	4 (7%)	49 (12.8%)	15 (22.7%)
31–35	92 (29.1%)	8 (14 0%)	54 (14.1%)	6 (9.1%)
36–40	93 (29.4%)	13 (22.8%)	46 (12.0%)	16 (24.2%)
41–45	38 (12.0%)	11 (19.3%)	45 (11.7%)	6 (9.1%)
46 and over	4 (1.3%)	-	-	8 (12.1%)
46–50		10 (17.5%)	44 (11.5%)	
51–55		3 (5.3%)	50 (13.1%)	
56–60		6 (10.5%)	35 (9.1%)	
61–65		2 (3.5%)	22 (5.7%)	
66–70		-	6 (1.6%)	
Gender				
Female		50 (87.7%)	377 (98.4%)	64 (97 0%)
Male		7 (12.3%)	4 (1.0%)	1 (1.5%)
Non-binary / rather not say			2 (0.6%)	1 (1.5%)
Identify as				
Aboriginal / Torres Strait Islander	12 (3.8%)	0	12 (3.4%)	5 (7.4%)
Neither	301 (96.2%)	55 (100 0%)	364 (96.8%)	63 (92.6%)
Highest completed education level				
Postgrad qualification	142 (44.8%)	48 (84.2%)	213 (55.6%)	15 (22.7%)
Bachelor degree	113 (35.6%)	7 (12.3)	153 (39.9%)	34 (51.5%)
Diploma	17 (5.4%)	2 (3.5%)	11 (2.9%)	34 (6.1%)
Certificate	24 (7.6%)	0	1 (0.3%)	3 (4.5%)
High school	16 (5.0%)	0	5 (1.3%)	7 (10.6%)
Early exit high school	5 (1.6%)	0	0	3 (4.5%)
Main language spoken at home				
English	307 (97.5%)	55 (96.5%)	357 (97%)	62 (93.9%)
Other	8 (2.5%)	2 (3.5%)	11 (3%)	4 (6.1%)
Main setting				
Urban	224 (72.5%)	42 (79.2%)	273 (71.7%)	55 (85.9%)
Regional/rural/remote	85 (27.5%)	11 (20.8%)	108 (28.4%)	9 (14.1%)
Ever tested positive for COVID-19?				
Yes	2 (0.6%)	1 (1.8%)	4 (1 0%)	0
No	198 (62.5%)	49 (86 0%)	310 (81.4%)	50 (75.8%)
Not applicable, never been tested	117 (36.9%)	7 (12.3%)	67 (17.6%)	16 (24.2%)
Currently pregnant				
Yes	60 (19.2%)	2 (3.7%)	20 (5.2%)	1 (1.6%)
No	253 (80.8%)	52 (96.3%)	362 (94.8%)	63 (98.4%)
Currently breastfeeding				
Yes	97 (32.8%)	8 (26.7%)	27 (7 0%)	3 (5.2%)
No	199 (67.2%)	22 (73.3%)	356 (93 0%)	55 (94.8%)

Respondents indicated their current vaccination status; those who had not yet been vaccinated were asked about their intentions to be vaccinated once the vaccine was available to them. Significantly more doctors had already been vaccinated compared with all other groups, and significantly more midwives had been vaccinated compared with women and midwifery students (p<0.001). Doctors and midwives were significantly more likely to respond that they intended to have the vaccine, and significantly more women indicated that they were not intending to be vaccinated (p<0.001) (Table 3).

**Table 3:** Current vaccination status and intentions for COVID-19 vaccination.

Vaccination status	Women	Medical practitioners	Midwives	Midwifery students
	N = 326	N = 58	N = 391	N = 78
I have already been vaccinated against COVID-19				
Yes	44 (14.4%)^a^	26 (49.1%)^c^	127 (33.4%)^b^	7 (10.9%)^a^
No	262 (85.6%)	27 (50.9%)	253 (66.6%)	57 (89.1%)
I will have the COVID-19 vaccine when it becomes available to me				
Yes (definitely and probably)	123 (48.0%)^a^	24 (88.9%)^b^	138 (57.7%)^c^	31 (62.0%)^a,b^
I am not sure	45 (17.6%)^a,b,c^	1 (3.7%)^c^	45 (18.8%)^b^	11 (22.0%)^a,c^
No (definitely and probably)	88 (34.4%)^a^	2 (7.4%)^b^	56 (23.4%) ^b^	8 (16.0%)^b^
I have been offered the COVID-19 vaccine but declined				
No	83 (32.5%)	15 (55.6%)	67 (28.4%)	16 (32.0%)
Yes	42 (16.5%)	4 (14.8%)	60 (25.4%)	3 (6.0%)
Not offered yet	128 (50.2%)	8 (29.6%)	109 (46.2%)	31 (62.0%)
The COVID-19 vaccines are safe [for myself]				
Agree	207 (75.3%)	47 (97.9%)	274 (81.8%)	45 (84.9%)
Disagree	68 (24.7%)	1 (2.1%)	61 (18.2%)	8 (15.1%)
The COVID-19 vaccines are effective [for myself]				
Agree	216 (78.5%)	48 (100%)	275 (82.6%)	46 (86.8%)
Disagree	59 (21.5%)	0	58 (17.4%)	7 (13.2%)

Each superscript letter denotes a subset of group categories whose column proportions do not differ significantly from each other (p<0.001).

Midwives and midwifery students had significantly higher levels of uncertainty regarding intentions to be vaccinated compared to all other groups. Although not statistically significant, more midwives had declined an offer for vaccination (p = 0.008) and fewer women perceived that the vaccine was safe (p = 0.002) or effective (p = 0.003) for them compared with all other cohorts.

We were interested in where people derived their information about the COVID-19 vaccine and whether they had enough information (Table 4). Almost three quarters of women (71%) perceived that they had enough information, while a higher proportion of doctors (80%), but fewer midwives (46%), and midwifery students (33%) felt that women in their care were provided with adequate information. The proportion of doctors agreeing with this statement was significantly higher than the proportions of midwives, midwifery students and women (p<0.001). Whilst ‘family and friends’ was given as a response option, this influence was not featured in the top three ranking for any of the cohorts’ responses.

**Table 4:** Sources of information regarding the COVID-19 vaccine and perceived value.

	Women	Medical practitioners	Midwives	Midwifery students
	N = 326	N = 58	N = 391	N = 78
**Top three most frequently accessed sources of information regarding the COVID-19 vaccine**				
1st	Health Professional Organisations websites 68%	University 60%	Newspapers 60%	Websites 44%
2nd	Social Media 46%	Websites 59%	Websites 49%	Social media 40%
3rd	Health Professionals (midwives, doctors) 42%	Journals 55%	Mainstream media 37%	Mainstream media 36%
**Perceptions about information provided to women to facilitate decision making regarding COVID-19 vaccination**				
I received [woman] /or the women I see in practice are provided with adequate information to make a decision whether to have the COVID-19 vaccine	214 (71.3%)^a^	41(80.4%)^a^	161 (45.5%)^b^	19 (33.4%)^b^

Each superscript letter denotes a subset of group categories whose column proportions do not differ significantly from each other (p<0.001).

Health care practitioners were asked about their recommendations regarding COVID-19 vaccination for childbearing women (Table 5). Doctors and midwifery students were significantly more likely to recommend the vaccine to pregnant women in their care than midwives (p<0.001). For any women of childbearing age, doctors were significantly more likely to recommend and less likely to be unsure about the vaccine for this group than midwives or midwifery students (p<0.001). Midwives were significantly more likely to recommend that women should wait until they were not pregnant to be vaccinated, while doctors were more likely to disagree with this recommendation (p<0.001). Significantly more doctors (80%) felt that women should not wait until breastfeeding had concluded before being vaccinated compared with 34% of midwives and 27% of midwifery students (p<0.001).

**Table 5:** Practices and attitudes regarding vaccination of women against COVID-19.

Practices and Attitudes	Medical practitioners	Midwives	Midwifery students
I would recommend to pregnant women to have the vaccine			
Yes	9 (18.4%)^b^	21 (6.1%)^a^	10 (17.9%)^b^
Not sure	10 (20.4%)^b^	122 (35.3%)^a^	24 (42.9%)^a^
No	30 (61.2%) ^a^	202 (58.7%) ^a^	22 (39.3%) ^b^
I am currently recommending all women of childbearing age to have the vaccine			
Yes	36 (70.6%) ^b^	131 (36.7%) ^a^	16 (28.6%) ^a^
Not sure	3 (5.9%) ^b^	123 (34.5%) ^a^	20 (35.7%) ^a^
No	12 (23.5%) ^a^	1023(28.9%) ^a^	20 (35.7%) ^a^
I think women should wait until they are not pregnant before having the COVID-19 vaccine			
Yes	22 (43.1%) ^a,b^	195 (54.6%) ^a^	21 (37.5%) ^b^
Not sure	12 (23.5%) ^b^	128 (35.9%) ^a^	26 (46.4%) ^a^
No	17 (33.3%) ^b^	34 (9.56%) ^a,b^	9 (16.1%) ^a^
I think that women should wait until they have concluded breastfeeding before having the COVID-19 vaccine			
Yes	1 (2.0%) ^b^	87 (24.4%) ^a^	12 (21.4%) ^a^
Not sure	9 (17.6%) ^b^	148 (41.5%) ^a^	29 (51.8%) ^a^
No	41 (80.4%) ^b^	122 (34.2%) ^a^	15 (26.8%) ^a^
I have concerns about the COVID-19 vaccine for the women I see in my care			
Yes	18 (35.3%)	188 ^b^ (52.8%)	26 (46.4%)
No	33 (64.7%)	168 (47.2%)	30 (53.6%)

Each superscript letter denotes a subset of group categories whose column proportions do not differ significantly from each other (p<0.001).

Finally, we asked about the factors that influenced participants’ decision to be vaccinated once it was available to them (Table 6). A desire to resume international travel motivated respondents broadly, as did a sense of duty to contribute to ending the pandemic, setting a good community example, and reducing exposure for children who are not currently covered in the Australian COVID-19 vaccination plan.

**Table 6:** Factors influencing decision to be vaccinated.

Influenced decision to be vaccinated when available	Women	Medical practitioners	Midwives	Midwifery students
I am employed or in clinical placement in an occupation that has increased risk for exposure to COVID-19				
Influential	129 (82.7%)	43 (97.7%)	235 (86.7%)	35 (89.7%)
Not influential	27 (17.3%)	1 (2.3%)	36 (13.3%)	4 (10.3%)
*Not applicable*	*107*	*4*	*43*	*10*
My partner is employed in an occupation that has increased risk for exposure to COVID-19				
Influential	59 (54.6%)^a^	22 (88%) ^b^	50 (32.3%) ^c^	9 (42.9%) ^a,b^
Not influential	49 (45.4%)	3 (12%)	105 (67.7%)	12 (57.1%)
*Not applicable*	*154*	*23*	*159*	*28*
My close friends or extended family are employed in an occupation that has increased risk for exposure to COVID-19				
Influential	85 (59.9%)	17 (58.6%)	87 (27.8%)	20 (58.8%)
Not influential	57 (40.1%)	12 (41.4%)	115 (36.7%)	14 (41.2%)
*Not applicable*	*120*	*19*	*111*	*15*
I have other conditions that increase my risk if I contract COVID-19				
Influential	48 (41.7%)	9 (47.4%)	74 (48.4%)	12 (44.4%)
Not influential	67 (58.3%)	10 (52.6%)	79 (51.6%)	15 (55.6%)
*Not applicable*	*146*	*29*	*161*	*22*
My partner has other medical conditions that increase their risk if they contract COVID-19				
Influential	26 (27.7%)	7 (41.2%)	43 (35%)	7 (38.9%)
Not influential	68 (72.3%)	10 (58.8%)	80 (65%)	11 (61.1%)
*Not applicable*	*167*	*31*	*192*	*31*
My close friends or family have other medical conditions that increase their risk if they contract COVID-19				
Influential	115 (69.7%)	20 (71.4%)	156 (72.6%)	25 (78.1%)
Not influential	50 (30.3%)	8 (28.6%)	59 (27.4%)	7 (21.9%)
*Not applicable*	*99*	*20*	*100*	*17*
I have friends and family overseas that I want to travel to see				
Influential	114 (65.5%)	22 (75.9%)	149 (65.1%)	26 (74.3%)
Not influential	60 (34.5%)	7 (24.1%)	80 (34.9%)	9 (25.7%)
*Not applicable*	*88*	*18*	*86*	*14*
I need to resume international travel for work				
Influential	25 (31.3%)	9 (45%)	20 (20.8%)	4 (23.5%)
Not influential	55 (68.8%)	11 (55%)	76 (79.2%)	13 (76.5%)
*Not applicable*	*182*	*28*	*219*	*32*
I want to resume international travel for leisure				
Influential	149 (70%)	31 (73.8%)	202 (73.5%)	29 (69%)
Not influential	64 (30%)	11 (26.2%)	73 (26.5%)	13 (31%)
*Not applicable*	*49*	*5*	*40*	*7*
I have a sense of duty to increase the chances of finishing the global pandemic				
Influential	177 (78.3%)^a^	43 (93.5%) ^b^	258 (88.7%) ^b^	39 (81.3%) ^a,b^
Not influential	49 (21.7%)	3 (6.5%)	33 (11.3%)	9 (18.8%)
*Not applicable*	*34*	*2*	*23*	*1*
I want to set a good example in my community				
Influential	174 (76.3%)	41 (89.1%)	239 (81.8%)	37 (77.1%)
Not influential	54 (23.7%)	5 (10.9%)	53 (18.2%)	11 (22.9%)
*Not applicable*	*34*	*2*	*24*	*1*
I want to be vaccinated to reduce the risk of exposing my children who are too young to be vaccinated				
Influential	152 (79.6%)	24 (82.8%)	113 (70.2%)	18 (66.7%)
Not influential	39 (20.4%)	5 (17.2%)	48 (29.8%)	9 (33.3%)
*Not applicable*	*71*	*19*	*155*	*22*

This is the first study to explore the perceptions and intentions regarding COVID-19 vaccination of both those who receive and provide maternity care in Australia. There is increasing recognition of the risk to women who are pregnant and who might become infected with SARS-CoV-2. Evidence is emerging of increased risk of stillbirth, preterm birth, and maternal and neonatal admissions to intensive care for pregnant women with COVID-19 [25]. In addition, early findings reveal that there may be even greater risk to pregnant women and younger populations from the new and emerging COVID-19 variants of concern [26]. It is timely therefore, to consider the guidelines, public health messaging and other factors that may influence women’s and health professionals’ preparedness to become vaccinated against COVID-19 in a country with low COVID-19 infection and death rates.

Most people living in Australia have enjoyed the health and social benefits of a broadly well-executed public health plan, supported by measures such as, strict international border controls, lock-down, mandatory mask-wearing, and various other measures that have managed the spread of COVID-19 within the community. [27]. Findings from our study add to the results of earlier research in general populations [14, 15] by revealing the perceptions and intentions regarding the COVID-19 vaccine specifically from the perspectives of consumers and providers of maternity care. A comparison of findings about the adequacy of information received or provided highlights a communication gap, which needs to be addressed to ensure consistent, clear and reinforced vaccination information is available.

At the time of the study, national guidelines indicated that breastfeeding women should consider being vaccinated against COVID-19 [2]. Despite this, more than half (54%) of the practitioners in our study were either ‘not sure’ or would not recommend the COVID-19 vaccination to women who were breastfeeding. This evidence of practitioner delay or hesitancy in aligning clinical practice with guideline recommendations is important especially considering the recent update to national guidelines recommending routine immunisation for all pregnant and breastfeeding women [5]. Future health messaging must target not only the public but importantly, the health professionals responsible for routine maternity care. Addressing practitioner hesitancy must be performed in parallel campaigns and should focus on providing resources to communicate the current safety data to their patients.

Healthcare professionals play an important role in influencing decision-making around vaccination [10, 28]. The mixed responses regarding intentions to be vaccinated from the professional cohort in our study who were already eligible for vaccination, points to the need for further professional and public health messaging to provide timely information to enable health professionals to participate in vaccination offered to them with confidence. Improved practitioner confidence and vaccination uptake is likely to influence supportive conversations with women [9, 29]. As vaccination programs around the world continue, observational safety data on the use of vaccines in pregnant and breastfeeding populations is mounting, with evidence indicating that the vaccine is safe in pregnancy [6]. There is now at least one US-based phase 2/3 clinical trial that is recruiting women to a randomised, placebo-controlled, observer blind study (NCT04754594) [7] which will provide more information in the future.

The second field of influence is the impact that childbearing women have on vaccine uptake, not only for their children but for the broader community, a phenomenon known as ‘cocoon immunisation’. This was exemplified in the Bordetella Pertussis outbreak in Australia during 2012, where the risk to newborns was increased following exposure from grandparents and extended family members, whose immunisation status had lapsed. Targeted public health messaging to expectant parents resulted in an increase in pertussis vaccination for older Australians who were directly influenced by new parents, who were restricting visits to the new baby to those with a current immunisation status [30, 31]. Public health campaigns encouraging COVID-19 vaccination should include new and expectant parents, realising the exponential potential for positive influence on the broader community including older people who are at increased risk if infected with COVID-19.

Two studies in 2020 that surveyed adults in Australia reported a 10 percent drop in intentions to be vaccinated against COVID-19 between April and June of that year, which was thought to be associated with the perception of reduced risk of contracting COVID [14, 15]. Since these studies were conducted, there has been further improvement in the understanding of management, and minimisation of community spread, of COVID-19, which has resulted in a diminishing in the risk of potential exposure to the virus in many parts of Australia. The external signals that indicate reduced risk of COVID-19 are increasing; these include less frequent lock-downs with scaled restrictions for shorter periods, schools and universities open, and infrequent interstate quarantine border controls. This comparative easing of restrictions to the activities of everyday life are closely linked to public perceptions of risk and threat and therefore, may influence individuals’ desire or intention to be vaccinated against COVID-19. This is consistent with our study findings, where the perception of choice, freedom, and the privilege of choosing between the risk of contracting COVID-19 compared with the risk of vaccination allows for a much higher threshold in those considering being vaccinated. Public responsiveness to vaccination is undeniably linked to the perception of risk of contracting COVID-19 which is susceptible to rapid and strong fluctuation in the current changing environment.

The evidence regarding factors that influence decisions to become vaccinated provides useful insights into factors that might influence or motivate consumers and providers of maternity care to be vaccinated. Data from this study gives unique understanding of the factors that might influence women who are currently pregnant, breastfeeding, or planning a pregnancy. Factors that influence vaccination intentions of health practitioners working in maternity are also novel. The valuable evidence provided in our findings may support targeted public health campaigns to increase vaccine uptake by these discrete cohorts. There is a pressing need for a well-coordinated and targeted public health communications campaign to encourage COVID-19 vaccination in Australia, as has occurred in New Zealand and Singapore. Distribution of clear and consistent messaging that the COVID-19 vaccine is safe, effective and recommended for pregnant people is urgently needed. If social incentives, such as, seeing friends and family and resuming usual activities, prove not to be sufficient, the use of financial incentives may help increase vaccination rates [32]. Findings may be used to support targeted public health campaigns to increase vaccine uptake by these discrete but connected maternity cohorts.

### Strengths and limitations

The strengths of this study are found in the range of stakeholder groups responded. The interconnectedness of practitioner support and women’s uptake of vaccines is well established. Surveying each of these key cohorts simultaneously adds strength to the survey findings that provide a snapshot of maternity consumer and provider perceptions and intentions at the same time. The timely collection and analysis of data obtained three months into the national vaccine roll-out is a benefit of this study. Findings will allow targeted and specific public health messaging to reach each group with an understanding of the perceptions of these unique and influential population groups.

The use of non-random convenience sampling is a limitation of this research. The rationale for this approach was to reduce the burden to a population with recognised stress in receiving or providing maternity care during a global pandemic. Our study was completed by participants who mostly spoke English at home; and who had attained higher education, readers should carefully consider the transferability of these findings to non-English speaking groups and those with potential disadvantage such as those with literacy, technology and English language challenges. The number of responses from women’s partners was also too low to include so the perception of this potentially influential group is unknown.

## Conclusion

This study has provided the first-known evidence of maternity stakeholder perceptions and intentions regarding the COVID-19 vaccine in Australia. Understanding the perspectives of pregnant and or breastfeeding women, those planning pregnancy and their care providers provides vital information for public health clinicians, policy makers, and those responsible for health service delivery as they plan, lead, and implement the national COVID-19 vaccine strategy in Australia. Previous evidence indicates the significance and importance of health practitioner engagement and recommendation of vaccination in the success of public health vaccination programs. The novel findings in this study will facilitate targeted and specific ‘wrap around’ public health messaging to further engage with each maternity stakeholder group to improve COVID-19 vaccine uptake.

## Supporting information

S1 File S1 Supplementary_CovMatVax survey. CovMat-Vax: A pulse check survey.
(DOCX)
