# Supplementary material for: COVID-19 vaccination perceptions and intentions of maternity care consumers and providers in Australia
Source: PLoS One. 2021 Nov 15;16(11):e0260049. doi: 10.1371/journal.pone.0260049 (PMC8592457; doi:10.1371/journal.pone.0260049)
Supplement: S1 File — CovMat-Vax: A pulse check survey. (DOCX) [file pone.0260049.s001.docx]

S1 File. S1 Supplementary_CovMatVax Survey. CovMat-Vax: A pulse check survey.

**CovMat- Vax: A pulse check Survey**

**Single survey link with front matter**

Thank you for your interest in this research which aims to explore individuals' perspectives and intentions regarding the COVID-19 vaccine from

- **women** of childbearing age and their **partners** and
- **midwives, doctors and midwifery students** providing maternity care in Australia.

To be eligible to participate in this study, you will need to meet one of the inclusion criteria below. Participation in this survey is voluntary, full study information is available here (hyperlinks to full PIF pdf once approved). You may withdraw at any time without consequence. If at any stage you wish to discontinue you may do so. Any data already entered may be used for analysis- owing to the anonymous nature of the survey, entries cannot be identified or, therefore, withdrawn.

It is possible to return to saved data and complete the survey within 14 days of commencing it providing you use the same device.

If you have any questions about this study, you may contact the principal investigator

If you would like to talk to someone about any feelings that have risen for you whilst participating in this study you can contact the National COVID-19,  24/7 Phone Counselling  Service on 1300 22 46 36.

**Consent > force answer**

- I confirm that I am 18 years or over, eligible and give my consent to participate in this survey

I am

- A woman in Australia of childbearing age
- A partner of a woman in Australia who is of childbearing age
- A midwife working in any setting in Australia
- A medical practitioner working with childbearing women in Australia
- A midwifery student working in any setting in Australia

Answer determines ‘stream’ of questions participants are asked.

All streams are prefaced with the Demographics questions

**Demographics**

**Tell us a little about yourself**

1. State live in + Gender for all other cohorts outside of women
2. Are you of Aboriginal and/ or Torres Strait Islander origin?
3. Do you speak a language other than English at home? IF Y please describe
4. Education Level
5. Age groupings (5 year sets) (RB) 18 – 25, 26 – 30, 31 -35, 36 – 40, 41– 45, 46+
6. Have you ever tested positive forCOVID-19? Y/N / NA not tested
7. Do you have family/ friends living outside of Australia? Y/N
8. Do you normally undertake international travel for work or holidays? Y/N
9. Has a family member or close friend in Australia tested positive for COVID-19? Y/N
10. Has a family member or close friend overseas tested positive for COVID-19 Y/N/ NA

**Individual Context**

|  |  |  |  |  |  |
| --- | --- | --- | --- | --- | --- |
|  | **Women** | **Partners** | **Midwives** | **Doctors** | **Midwifery Students** |
| 1 | Currently pregnant – weeks N/ Y – 4 -43 weeks DD | Is your partner currently pregnant N/ Y  4 -43 weeks DD | Currently pregnant – weeks N/ Y – 4 -43 weeks DD | Currently pregnant – weeks N/ Y – 4 -43 weeks DD | Currently pregnant – weeks N/ Y – 4 -43 weeks DD |
| 2 | Currently breastfeeding N/ Y age of baby  Months up to 24 DD | Currently breastfeeding N/ Y age of baby | Currently breastfeeding N/ Y age of baby | Currently breastfeeding N/ Y age of baby | Currently breastfeeding N/ Y age of baby |
|  | Planning pregnancy in the next **2 years** Y/N | Planning pregnancy in the next 2 years | Planning pregnancy in the next 2 years | Planning pregnancy in the next 2 years | Planning pregnancy in the next 2 years |
| 3 |  |  | How long have you been a midwife? | How long been a doctor? | What Midwifery Course are you currently enrolled in? (DD) B Mid, Grad Dip, Master, Dual degree |
| 4 | What area do you live in?  Urban  Regional  Rural  Remote | What area do you live in?  Urban  Regional  Rural  Remote | What area do you mainly work in?  Urban  Regional  Rural  Remote | What area do you mainly work in?  Urban  Rural  Regional  Remote | What area do you mainly work in?  Urban  Rural  Regional  Remote |
| 5 | Are you employed in a Phase 1A occupation for COVID-19 vaccine access? Y/ N  If Y please indicate  Health care worker  Quarantine worker (hotel, sea/air ports)  Aged care worker | Are you employed in a Phase 1A occupation for COVID-19 vaccine access? Y/ N  If Y please indicate  Health care worker  Quarantine worker (hotel, ports)  Aged care worker | Please indicate which model you mainly work in  Standard Public Hospital  Midwifery Group Practice  Private obstetric  Primary care setting  Privately Practising Midwife  Midwifery education/ research | Please indicate main health setting  General Practice  Hospital practice  Public Health  Private specialist practice  Type of doctor – GP, trainee, specialist… |  |
| 6 | Have you had a discussion with a HCP about receiving the Covid-19 Vaccination for yourself  If Y  Midwife  Doctor  Child Health Nurse  Have you had a discussion with your HCP re Covid-19 Vaccination for your partner?  Yes – no – Not sure (NS) | Have you had a discussion with a HCP about receiving the Covid-19 Vaccination for yourself  Have you had a discussion with your HCP re Covid-19 Vaccination for your partner?  Yes – no – NS | With the woman in your care, are you discussing Covid-19 Vaccination?  Often – sometimes – rarely – never (not applicable as I am not providing clinical care) | With the woman in your care, are you discussing Covid-19 Vaccination  Often – sometimes – rarely – never (not applicable as I am not providing clinical care | With the woman in your care, have you seen HCPs discussing Covid-19 Vaccination?  Often – sometimes – rarely-never |
| 7 | Where have you obtained information about Covid-19 vaccination (tick all that apply  Midwife  Doctor  Nurse  Facebook  Twitter  … Reditt  Instagram  …. You-tube  Snapchat  ….Other social media  Websites (government - national or state health departments)  Health professional organisation websites (RANZCOG, ACM)  Websites (personal blogs)  Newspapers (online, apps or paper based)  Published journal articles  Family members  Friends  Colleagues/ workplace  Other - specify | Where have you obtained information about Covid-19 vaccination (tick all that apply)  Midwife  Doctor  Nurse  Social media (personal network)  Social media (professional sites)  Websites (government national or state health departments)  Websites (personal blogs)  Newspapers (online apps or paper based)  Published journal articles  Family members  Friends  Colleagues  Employer  Other - specify | Where have you obtained information about Covid-19 vaccination (tick all that apply)  Social media  Websites (government national or state health departments)  Health professional organisation websites (RANZCOG,RACGP, ACM)  Websites (personal blogs)  Newspapers (online, apps or paper based)  Published journal articles  Family members  Friends  Colleagues  Employer  Other - specify | Where have you obtained information about Covid-19 vaccination (tick all that apply)  Social media  Websites (government national or state health departments)  Health professional organisation websites (RANZCOG,RACGP, ACM)  Websites (personal blogs)  Newspapers (online, apps or paper based)  Published journal articles  Family members  Friends  Colleagues/ workplace  Other - specify | Where have you obtained information about Covid-19 vaccination (tick all that apply)  Social media  Websites (government national or state health departments)  Health professional organisation websites (RANZCOG,RACGP, ACM)  Websites (personal blogs)  Newspapers (online, apps or paper based)  Published journal articles  Family members  Friends  Colleagues/ workplace  University  Other - specify |

**About the Vaccine**

|  | **Women** | **Partners** | **Mid** | **Doc** | **Mid St** |
| --- | --- | --- | --- | --- | --- |
| **ANSWER THESE QUESTIONS ABOUT THE COVID-19 VACCINE IN GENERAL** | | | | | |
| 8 | I feel like I have adequate information to make a decision about receiving the COVID-19 vaccine  Strongly Agree, Agree, Somewhat Disagree, Disagree, Strongly Disagree | I feel like I have adequate information to make a decision about receiving the COVID-19vaccine  Strongly Agree, Agree, Somewhat Disagree, Disagree, Strongly Disagree | I feel like the women in my care are provided with adequate information to make a decision about whether to have the COVID-19 vaccine  Strongly Agree, Agree, Somewhat Disagree, Disagree, Strongly Disagree | I feel like the women in my care are provided with adequate information to make a decision about whether to have the COVID-19 vaccine  Strongly Agree, Agree, Somewhat Disagree, Disagree, Strongly Disagree | I feel like the women I see in practice are provided with adequate information to make a decision about whether to have the COVID-19 vaccine  Strongly Agree, Agree, Somewhat Disagree, Disagree, Strongly Disagree |
| 9 | I have been recommended to have the COVID 19 vaccine – Y / N | I have been recommended to have the COVID 19 vaccine – Y / N | I am recommending to pregnant women to have the vaccine – Y / N  I am recommending all women of childbearing age have the vaccine – Y/N  If NO –: please clarify FREE TEXT | I am recommending to pregnant women to have the vaccine – Y / N  I am recommending all women of childbearing age have the vaccine – Y/N  If NO – please clarify FREE TEXT | I would recommend to pregnant women to have the vaccine – Y / N  I am recommending all women of childbearing age have the vaccine – Y/N  If NO – please clarify: FREE TEXT |
| 10 | I will have the vaccine when it becomes available to me  Definitely yes  Probably yes  I am not sure  Probably no  Definitely no | My partner will have the vaccine when it becomes available  Definitely yes  Probably yes  I am not sure  Probably no  Definitely no | I will have the vaccine when it becomes available to me  Definitely yes  Probably yes  I am not sure  Probably no  Definitely no | I will have the vaccine when it becomes available to me  Definitely yes  Probably yes  I am not sure  Probably no  Definitely no | I will have the vaccine when it becomes available to me  Definitely yes  Probably yes  I am not sure  Probably no  Definitely no |
|  |  |  |  |  |  |
| 11 |  |  | I think that women should wait until they are not pregnant before having the COVID-19 vaccine – Y/N | I think that women should wait until they are not pregnant before having the COVID-19 vaccine – Y/N | I think that women should wait until they are not pregnant before having the COVID-19 vaccine – Y/N |
| 12 |  |  | I think that women should wait until they have concluded breastfeeding before having the COVID-19 vaccine – Y/N | I think that women should wait until they have concluded breastfeeding before having the COVID-19 vaccine – Y/N | I think that women should wait until they have concluded breastfeeding before having the COVID-19 vaccine – Y/N |
| 13 | I have concerns about the COVID-19 vaccine  Y/N  If Yes  concerned about future fertility risks  concerned about safety for my baby  concerned about my personal safety  Other: FREE TEXT | I have concerns about the COVID-19 vaccine  Y/N  If Yes  concerned about future fertility risks  concerned about safety for my baby  concerned about my personal safety  Other: FREE TEXT | I have concerns about the COVID-19 vaccine  Y/N  If Yes  concerned about my personal future fertility risks  concerned about the risks to fertility for women in my care  concerned about safety for my baby  Concerned about the safety for the babies in my care  concerned about my personal safety  Concerned about the safety of women in my care  Other: FREE TEXT | I have concerns about the COVID-19 vaccine  Y/N  If Yes  concerned about my personal future fertility risks  concerned about the risks to fertility for women in my care  concerned about safety for my baby  Concerned about the safety for the babies in my care  concerned about my personal safety  Concerned about the safety of women in my care  Other: FREE TEXT | I have concerns about the COVID-19 vaccine  Y/N  If Yes  concerned about my personal future fertility risks  concerned about the risks to fertility for women in general  concerned about safety for my baby  Concerned about the safety for the babies I see in practice  concerned about my personal safety  Concerned about the safety of women I see in practice  Other: FREE TEXT |
|  |  |  |  |  |  |
|  |  |  |  |  |  |
| RESPOND TO THESE QUESTIONS ABOUT THE VACCINE FOR YOU | | | | | |
| 14 | The COVID-19 vaccines are safe  Strongly Agree, Agree, Somewhat Disagree, Disagree, Strongly Disagree  NS | The COVID-19 vaccines are safe  Strongly Agree, Agree, Somewhat Disagree, Disagree, Strongly Disagree  NS | The COVID-19 vaccines are safe  Strongly Agree, Agree, Somewhat Disagree, Disagree, Strongly Disagree  NS | The COVID-19 vaccines are safe  Strongly Agree, Agree, Somewhat Disagree, Disagree, Strongly Disagree  NS | The COVID-19 vaccines are safe  Strongly Agree, Agree, Somewhat Disagree, Disagree, Strongly Disagree  NS |
| 15 | The COVID-19 vaccines are effective  Strongly Agree, Agree, Somewhat Disagree, Disagree, Strongly Disagree  NS | The COVID-19 vaccines are effective  Strongly Agree, Agree, Somewhat Disagree, Disagree, Strongly Disagree  NS | The COVID-19 vaccines are effective  Strongly Agree, Agree, Somewhat Disagree, Disagree, Strongly Disagree  NS | The COVID-19 vaccines are effective  Strongly Agree, Agree, Somewhat Disagree, Disagree, Strongly Disagree  NS | The COVID-19 vaccines are effective  Strongly Agree, Agree, Somewhat Disagree, Disagree, Strongly Disagree  NS |
| 16 | I’ll wait till my pregnancy has finished then I will have vaccine  Yes No Not sure yet | My partner will wait until she has given birth and then will have the vaccine  Yes No Not sure yet | I have already been vaccinated against COVID-19  Yes No Prefer not to say | I have already been vaccinated against COVID-19  Yes No Prefer not to say | I have already been vaccinated against COVID-19  Yes No Prefer not to say |
| 17 | I’ll wait till I’ve finished BF and then I will have the vaccine | My partner will have the vaccine after she has finished breastfeeding | I have been offered the vaccine but declined  Yes No Prefer not to say  If Y please consider sharing your reasons | I have been offered the vaccine but declined  Yes No Prefer not to say  If Y please consider sharing your reasons | I have been offered the vaccine but declined  Yes No Prefer not to say  If Y please sharing your reasons |
| 18 | If you have already had the vaccine or you’ve decided to be vaccinated please rate how influential the following factors were for you in your decision to be vaccinated   - I am employed in an occupation that has increased risk for exposure to COVID-19 - My partner is employed in an occupation that has increased risk for exposure to COVID-19 - My close friends or extended family are employed in an occupation that has increased risk for exposure to COVID-19 - I have other conditions that increase my risk if I contract COVID-19 - My partner has other medical conditions that increase their risk if they contract COVID-19 - My close friends or family have other medical conditions that increase their risk if they contract COVID-19 - I have friends and family overseas that I want to travel to see - I need to resume international travel for work - I want to resume international travel for leisure - I have a sense of duty to increase the chances of finishing the global pandemic - I want to set a good example in my community - I want to be covered to reduce risk of exposing my children who are too young to be vaccinated - Other please explain   Very influential, somewhat influential, not influential, not at all influential, not applicable to me | If you have already had the vaccine or you’ve decided to be vaccinated please rate how influential the following factors were for you in your decision to be vaccinated   - I am employed in an occupation that has increased risk for exposure to COVID-19 - My partner is employed in an occupation that has increased risk for exposure to COVID-19 - My close friends or extended family are employed in an occupation that has increased risk for exposure to COVID-19 - I have other conditions that increase my risk if I contract COVID-19 - My partner has other medical conditions that increase their risk if they contract COVID-19 - My close friends or family have other medical conditions that increase their risk if they contract COVID-19 - I have friends and family overseas that I want to travel to see - I need to resume international travel for work - I want to resume international travel for leisure - I have a sense of duty to increase the chances of finishing the global pandemic - I want to set a good example in my community - I want to be covered to reduce risk of exposing my children who are too young to be vaccinated - Other please explain   Very influential, somewhat influential, not influential, not at all influential, not applicable to me | If you have already had the vaccine or you’ve decided to be vaccinated please rate how influential the following factors were for you in your decision to be vaccinated   - I am employed in an occupation that has increased risk for exposure to COVID-19 - My partner is employed in an occupation that has increased risk for exposure to COVID-19 - My close friends or extended family are employed in an occupation that has increased risk for exposure to COVID-19 - I have other conditions that increase my risk if I contract COVID-19 - My partner has other medical conditions that increase their risk if they contract COVID-19 - My close friends or family have other medical conditions that increase their risk if they contract COVID-19 - I have friends and family overseas that I want to travel to see - I need to resume international travel for work - I want to resume international travel for leisure - I have a sense of duty to increase the chances of finishing the global pandemic - I want to set a good example in my community - I want to be covered to reduce risk of exposing my children who are too young to be vaccinated   Very influential, somewhat influential, not influential, not at all influential, not applicable to me   - Other please explain | If you have already had the vaccine or you’ve decided to be vaccinated please rate how influential the following factors were for you in your decision to be vaccinated   - I am employed in an occupation that has increased risk for exposure to COVID-19 - My partner is employed in an occupation that has increased risk for exposure to COVID-19 - My close friends or extended family are employed in an occupation that has increased risk for exposure to COVID-19 - I have other conditions that increase my risk if I contract COVID-19 - My partner has other medical conditions that increase their risk if they contract COVID-19 - My close friends or family have other medical conditions that increase their risk if they contract COVID-19 - I have friends and family overseas that I want to travel to see - I need to resume international travel for work - I want to resume international travel for leisure - I have a sense of duty to increase the chances of finishing the global pandemic - I want to set a good example in my community - I want to be covered to reduce risk of exposing my children who are too young to be vaccinated - Other please explain   Very influential, somewhat influential, not influential, not at all influential, not applicable to me | If you have already had the vaccine or you’ve decided to be vaccinated please rate how influential the following factors were for you in your decision to be vaccinated  I am employed or in clinical placement in an occupation that has increased risk for exposure to COVID-19  My partner is employed in an occupation that has increased risk for exposure to COVID-19  My close friends or extended family are employed in an occupation that has increased risk for exposure to COVID-19  I have other conditions that increase my risk if I contract COVID-19  My partner has other medical conditions that increase their risk if they contract COVID-19  My close friends or family have other medical conditions that increase their risk if they contract COVID-19  I have friends and family overseas that I want to travel to see  I need to resume international travel for work  I want to resume international travel for leisure  I have a sense of duty to increase the chances of finishing the global pandemic  I want to set a good example in my community  I want to be covered to reduce risk of exposing my children who are too young to be vaccinated  Very influential, somewhat influential, not influential, not at all influential, not applicable to me  Other please explain |

Three words to describe your opinions about the COVID-19 vaccine during pregnancy / breastfeeding

Any further comments

Thank you for participating in this survey, your time is greatly appreciated.
